# Intermuscular Coordination in the Power Clean Exercise: Comparison between Olympic Weightlifters and Untrained Individuals—A Preliminary Study

**DOI:** 10.3390/s21051904

**Published:** 2021-03-09

**Authors:** Paulo D. G. Santos, João R. Vaz, Paulo F. Correia, Maria J. Valamatos, António P. Veloso, Pedro Pezarat-Correia

**Affiliations:** 1 Neuromuscular Research Lab, Faculty of Human Kinetics, Lisbon University, 1499-002 Cruz Quebrada-Dafundo, Portugal; pauloduarteguiasantos@gmail.com (P.D.G.S.); paulo.correia.performance@gmail.com (P.F.C.); mjvalamatos@fmh.ulisboa.pt (M.J.V.); ppezarat@fmh.ulisboa.pt (P.P.-C.); 2 CIPER, Faculty of Human Kinetics, Lisbon University, 1499-002 Cruz Quebrada-Dafundo, Portugal; apveloso@fmh.ulisboa.pt; 3 Biomechanics and Functional Morphology Laboratory, Faculty of Human Kinetics, Lisbon University, 1499-002 Cruz Quebrada-Dafundo, Portugal

**Keywords:** strength training, neural adaptations, muscle coordination, muscle synergies, electromyography

## Abstract

Muscle coordination in human movement has been assessed through muscle synergy analysis. In sports science, this procedure has been mainly applied to the comparison between highly trained and unexperienced participants. However, the lack of knowledge regarding strength training exercises led us to study the differences in neural strategies to perform the power clean between weightlifters and untrained individuals. Synergies were extracted from electromyograms of 16 muscles of ten unexperienced participants and seven weightlifters. To evaluate differences, we determined the pairwise correlations for the synergy components and electromyographic profiles. While the shape of activation patterns presented strong correlations across participants of each group, the weightings of each muscle were more variable. The three extracted synergies were shifted in time with the unexperienced group anticipating synergy #1 (−2.46 ± 18.7%; *p* < 0.001) and #2 (−4.60 ± 5.71%; *p* < 0.001) and delaying synergy #3 (1.86 ± 17.39%; *p* = 0.01). Moreover, muscle vectors presented more inter-group variability, changing the composition of synergy #1 and #3. These results may indicate an adaptation in intermuscular coordination with training, and athletes in an initial phase of training should attempt to delay the hip extension (synergy #1), as well as the upper-limb flexion (synergy #2).

## 1. Introduction

Regular practice of strength training is associated to increases in maximal strength, changes in neuromuscular function, and in muscle morphology. Neural adaptations to strength training occur earlier than muscle adaptations and the initial gains in strength are not accompanied by an increase in muscle size [1,2,3]. Furthermore, changes in the neural drive have been inferred from surface electromyography (EMG) studies that show increases in EMG activity of the agonist muscle during first weeks of training [1,2,3,4,5]. This increase in EMG reflects increases in fiber recruitment or firing frequency [1,4]. Additionally, motor unit recruitment thresholds are reduced in response to training [1,5,6]. Regarding neural adaptations in intermuscular coordination, three mechanisms are described to promote gains in strength performance: increased activation of agonists, decreased activation of antagonists, and activation of synergists. Thus, the coordination of all muscles would allow to produce the required joint moments in the intended direction to perform specific movements [1,4,6,7].

Olympic weightlifting (OWL) movements have been used in training programs developed to increase muscle strength and power in other sports. This evidence is mainly due to the relationship between OWL movement performance and other performance variables that may predict success in sports (like power, speed, agility or vertical jump height). OWL technique promotes the whole-body muscle recruitment at high speeds, while lifting high loads [8], and the complexity of the related exercises enables the understanding of the processes of muscle coordination. When untrained individuals perform whole-body and power movements, like in OWL, the level of activation of the antagonists may be greater compared to less complex movements [1,9]. Thus, it is essential that the nervous system is able to control unnecessary co-contraction of the antagonists, which may possibly result in a reduced power output [6,7,9,10]. After training, however, a reduced antagonist/agonist ratio of the knee joint in vertical jumps was observed in participants involved in an OWL protocol, when compared to traditional resistance training [9]. This possibly indicates increases in knee extension torque at propulsion, in contrast to the traditional resistance training group that exhibited a higher ratio of coactivation.

Despite the previously referred evidence on neural adaptations to training, there is scarce information about how the muscles are recruited and coordinated in order to perform strength training tasks. The quantification of muscle coordination has been studied through the decomposition of surface EMG recordings into muscle synergies. Each synergy integrates the combination of multiple muscles, and the combination of recruitment of multiple synergies will result in the production of a wide range of possible complex movements. Thus, specific tasks can be achieved by modulating neural commands that specify patterns of muscle activation, resulting in the production of natural motor behaviors [11,12]. The extraction of muscle synergies is typically obtained by means of the nonnegative matrix factorization (NMF) algorithm [13]. Briefly, this algorithm provides two components, which set information about timing of activation–activation coefficients and the weight of each muscle in each synergy–synergy vectors during movements [14,15].

In the sports sciences field, muscle synergies have been studied in walking and running [16], postural control tasks in response to unexpected external perturbations [17], pedaling [18,19], rowing [20], backward giant swing [21], breaststroke swimming [22], and bench press [23]. One of the topics that requires muscle synergy analysis is the characterization of the effect of training expertise in muscle coordination and the interindividual variability within highly skilled individuals. A comparison between highly trained female ice hockey players and non-athletes concluded that, during this kind of tasks, hockey players have shorter recovery periods in the center of mass stabilization and present less coactivation of muscle synergies [17]. During a rowing task, similar muscle synergies were extracted [20], while in breaststroke swimming, although muscle synergies were not profoundly affected when comparing experts and novices, differences in timing of activation of one extracted synergy and individual EMG profiles were found [22]. However, some accentuated differences between differently leveled groups have been found, namely during a badminton smash shot, where one particular synergy was found just in advanced players, revealing that enhanced performance in badminton smash shots may be related with neural strategies developed by training [24]. Moreover, interindividual variability in muscle synergies in highly trained cyclists [19], pole vaulters [25] or gymnasts [21] has been found. Particularly regarding strength training, Kristiansen and colleagues [23] have investigated muscle coordination in power lifters and untrained subjects, showing that powerlifters exhibited larger inter-subject variability in muscle vectors of concentric phase of bench press when compared to untrained subjects, corroborating these results with a five-week training protocol of untrained individuals [26]. Furthermore, four weeks of wobble board training appeared to modify modular organization in the early landing phase of a single-leg drop-landing with separation of the synergy comprising plantar flexors and ankle evertors, while the activation of secondary muscles was reduced [27]. 

Strength gains in advanced training stages are associated to continued muscle adaptations. However, the role of training in the refinement of strength training tasks and the possible timings of skill acquisition are issues that remain not sufficiently explored, although it is known that intermuscular coordination continues to adapt to prolonged resistance training [10]. Considering that Kristiansen and colleagues [26] provided the only study associating the extraction of muscle synergies and the neural strategies developed in a strength training process, we aimed to study a task with more degrees of freedom regarding strength training: the power clean exercise. The power clean is a variation of OWL exercises, where the high barbell velocities and loads request greater power outputs, demanding a coordinative strategy well defined by the practitioners. This exercise is commonly used for training and assessment of physical capacities and it has been previously shown to be a reliable indicator of performance in experienced [28], unexperienced [29], and adolescent athletes [30], while muscle synergies extracted from this exercise showed to be also reliable [31]. Thus, the present study aimed to investigate and compare the neural strategies underlying the power clean exercise between trained and untrained subjects. According to the previously presented evidence, we hypothesized that EMG profiles will present time-shifts regarding the activation coefficients, and that the weighting of the muscles within each synergy will present differences when comparing the two groups. We also intended to verify if weightlifters present variability regarding subject-specific adaptations. 

## 2. Materials and Methods

### 2.1. Participants

Seventeen male participants, 10 unexperienced (UNE; age 24.8 ± 2.0 years, height 173.2 ± 4.3 cm, body mass 68.6 ± 6.3 kg) and 7 experienced weightlifters (EXP; age 30.7 ± 9.3 years, height 177.6 ± 6.0 cm, body mass 85.9 ± 9.0 kg) with average five repetition maximum (5 RM) in power clean of 53.3 ± 9.8 kg and 102.1 ± 9.6 kg, respectively, participated in this study. The UNE participants were healthy physical education students without prior knowledge about the critical points of the exercise, while EXP were practitioners with at least two years of experience and at least 4 training sessions per week. All participants provided informed written consent. This study was approved by the Faculty’s Ethics Committee (CEFMH 4/2018) and all procedures adhered to the Declaration of Helsinki.

### 2.2. Experimental Approach

Each UNE participant performed two sessions, while EXP participants performed only one session. The first session of the UNE had the purpose of familiarize the participants with the task and with the laboratory equipment. In this familiarization, all UNE participants had a one-hour session of technical learning of the movement, with video demonstration and a first experience performing the exercise. The subsequent session with evaluation purposes for UNE participants (approximately one week after the familiarization). The only session performed by EXP participants was performed to evaluate the neural strategies associated to a complex strength training exercise, the power clean, and to assess intra- and intergroup variability of muscle synergies in UNE and EXP participants. 

In the evaluation session, the participants performed a warm-up followed by a 5 RM power clean test (as described in the following section); then, they had a period of recovery while the electrodes and the reflexive markers were placed. After this, the participants performed a re-warm-up, and then, one set of three repetitions with 90% of the 5 RM for EMG amplitude normalization, three sets of eight repetitions with 70% of the 5 RM for intra- and inter-group variability analysis.

### 2.3. Data Collection and Materials

To measure the displacement of the barbell, three reflexive markers were placed to each side of it, and an eight-camera system (Qualisys, Gothenburg, Sweden) was used to collect the movement data relative to the barbell displacement during the exercise. A sample rate of 200 samples/s was used for the movement data collection. Regarding to the myoelectric signals collection, sixteen muscles of the right side of the body were considered: upper trapezius (TS), pectoralis major (PM), biceps brachii (BB), triceps brachii lateral head (TB), flexor digitorum superficialis (FDS), extensor digitorum communis (EDC), latissimus dorsi (LD), erector spinae (ES), rectus abdominis (RA), external oblique (OE), gluteus maximus (Gmax), vastus lateralis of quadriceps (VL), biceps femoris long head (BF), semitendinosus (ST), lateral gastrocnemius (GL), and tibialis anterior (TA). We selected the muscles that would allow us to analyze the whole-body coordination, assessing pairs of agonist-antagonist muscles of the trunk, lower and upper limb. The barbell trajectory during the exercise did not allow us to place electrodes on anterior deltoideus and rectus femoris of the quadriceps. The electrodes placement followed the guidelines suggested by SENIAM (surface EMG for non-invasive assessment of muscles), excepting FDS, EDC, LD, PM, RA, and OE: FDS and EDC were placed according to Zipp [32]; the placement of LD followed the recommendations of Sèze and Cazalets [33]; PM was located medially to the anterior axillary border; RA and OE were both positioned laterally from the umbilicus, at 3 and 15 cm, respectively. To minimize skin impedance, the skin was shaved and cleaned with alcohol, and only after the electrodes were positioned according to the locations described above. For the acquisition of surface EMG signals, we used sixteen bipolar surface electrodes (EMG Delsys, Trigno^TM^), fixed to the skin with adhesive interface according to the orientation of the muscle fibers [34] and additionally fixed with tape avoiding possible artefacts resulting from the movement.

The power clean 5 RM test was performed after the warm-up. The 5 RM load was determined as the highest load the participants lifted five times, and the load of the last sets was increased by 2.5–5 kg, until the load corresponding to the 5 RM. Between each set, participants rested for four minutes, and after the test they had approximately one hour to recover during the placement of the EMG electrodes and reflexive markers. The participants were asked to perform a first of three repetitions with 90% of the 5 RM load. This set was used for task-specific submaximal dynamic normalization, despite the fact that there is debate about the amplitude normalization procedure while extracting muscle synergies [23]. After, the participants performed four sets of eight repetitions using 70% of the 5 RM load. The participants were instructed to perform the repetitions in a continued touch-and-go way, with an explosive ascendant phase and a controlled descendant phase. The movement phases were based on the barbell movement, with its lowest position when the disc are in contact with the ground defining the beginning of the ascendant phase, and its highest position representing the ending of the same phase. The same events were used oppositely to define the descendant phase. 

### 2.4. Data Processing

For the barbell phase definition, a time normalization from 0% to 100% was used to relativize each phase, since the same relative time phases may be different for different individuals. A low-pass filter (8 Hz, 4th order Butterworth) was used to smooth the barbell displacement that defined the phases. We excluded the first and last repetitions of each set.

For the pre-processing of the EMG data, firstly, we filtered using a band-pass filter (20–450 Hz), and then, we rectified and smoothed with a low-pass filter (12 Hz, 4th order Butterworth) each signal. Additionally, the signals were normalized to the average value of the 100 ms across the peak of the linear envelope of the set of 3 repetition with 90% of the 5 RM. Finally, and since the aim of this study was not to characterize the degree of muscle activation, we interpolated to 100 points each linear envelope [15].

### 2.5. Extraction of Muscle Synergies

For the extraction of muscle synergies, we implemented the algorithm proposed by Lee and Seung [13] for non-negative matrix factorization. This procedure minimizes the residual Frobenius norm between the initial matrix and the matrices resulting of its decomposition, which is given as:

*E = WC + e* *W ≥* 0 *^min^ ||E − WC||_FRO_* *C ≥ 0* (1)

where *E* is a *p*-by-*n* matrix (*p* = number of muscles; *n* = number of time points), *W* is a *p*-by-*s* (*s* = number of synergies), *C* is an *s*-by-*n* matrix, and *e* is a *p*-by-*n* matrix. ||.||*_FRO_* establishes the Frobenius norm and e is the residual error matrix. Thereby, the decomposition of the initial matrix resulted in two mulplication matrices corresponding to two different synergy components: the muscle synergy vectors (*W*), representing the weighting of each collected muscle within the extracted synergies, and the synergy activation coefficient (*C*), representing the activation of the synergies along the time of the movement. 

The algorithm was iterated 100 times by varying the number of synergies between 1 and 16. Four to eight repetitions of each set were analyzed. Therefore, E consisted in a 16 row by 800 to 1600 columns matrix. Variance accounted for (VAF) defined the number of muscle synergies. Thus, the number of synergies that defined 90% of VAF, as long as each synergy represented at least 5% of VAF, was the number of synergies selected for analysis. Additionally, we calculated VAF for each muscle (VAF_muscle_), which was used to ensure that the activity patterns of the muscle accounted the extracted muscle synergies. The extracted muscle synergies were manually sorted since the algorithm does not apply the same sorting way for all the sets of the participants.

### 2.6. Assessment of within- and between-Group Similarity

The maximum cross-correlation function (r_max_), which is an indicator of waveform similarity, was used to assess differences in individual EMG patterns and synergy activation coefficients. Simultaneously, we determined the lag times at the maximum cross correlation function using Matlab 2015a (Mathworks Inc., Natick, MA, USA) xcorr function for centered data (option “coeff”), which allowed to assess differences in timing of activation of muscles and synergies. The index of within- and between-group variability was assessed by averaging the r_max_-values of all the within- and between-group pairwise (70 untrained-weightlifter pairs (7 weightlifters × 10 untrained), 45 untrained pairs (each of the 10 participants compared with each of the other 9), and 21 weightlifters pairs (each of the 7 participants compared with each of the other 6)). The calculated indexes were used as indicators of the waveform consistency between and within groups [20,22]. Additionally, the r-values of the muscle synergy vectors were calculated for each pair of participants.

Additionally, similarity of muscle synergies within each group and between weightlifters and untrained participants was assessed by verifying if the extracted muscle synergies from all participants accounted for the EMG patterns of each untrained and weightlifter. The vectors matrix extracted from unexperienced participants (***W****untrained*) was held fixed in the algorithm while synergy activation coefficient (***C****participant*) of each compared participant varied. This component was initialized randomly and was iterated until convergence. The EMG data matrix of the compared participant (***E****participant*) was provided to the algorithm with the following update rule [13]:

(2) $${\left(Cparticipant\right)}_{ij}\;\leftarrow \;{\left(Cparticipant\right)}_{ij}\;\frac{{\left(Wcontrol\;Eparticipant\right)}_{ij}}{{\left(Wcontrol\;Wcontrol\;Cparticipant\right)}_{ij}}$$

The accuracy of the reconstructed individual EMG patterns was quantified by the overall VAF, using the fixed muscle vectors and the newly computed synergy activation coefficients.

### 2.7. Statistical Analysis

We verified normality through the Shapiro–Wilk test (SPSS version 25.0, SPSS Inc., Chicago, IL, USA) using a significance level of *p* < 0.05. A sample Student’s t-test with zero as reference value was used to evaluate the differences in the lag time within- and between-groups. On the other hand, a one-sample Wilcoxon signed rank test was used when normality was not assumed. An independent sample *t*-test was used to compare age, height, body mass, 5 RM load, VAF, VAF_muscle_, r-, and r_max_-values between groups, and as measure of effect size, we reported Cohen’s d. A Mann–Whitney U-test was used when normality was not assumed.

## 3. Results

Regarding the participants characteristics, no differences were shown between groups for age (*p* = 0.173) nor height (*p* = 0.120), while significant differences were found for body mass (*p* = 0.001; 68.6 ± 6.3 and 85.9 ± 9.0 kg for UNE and EXP, respectively) and 5 RM load (*p* < 0.001; 53.3 ± 9.8 kg and 102.1 ± 9.6 kg for UNE and EXP, respectively).

### 3.1. Muscle Synergies

**Figure 1:**
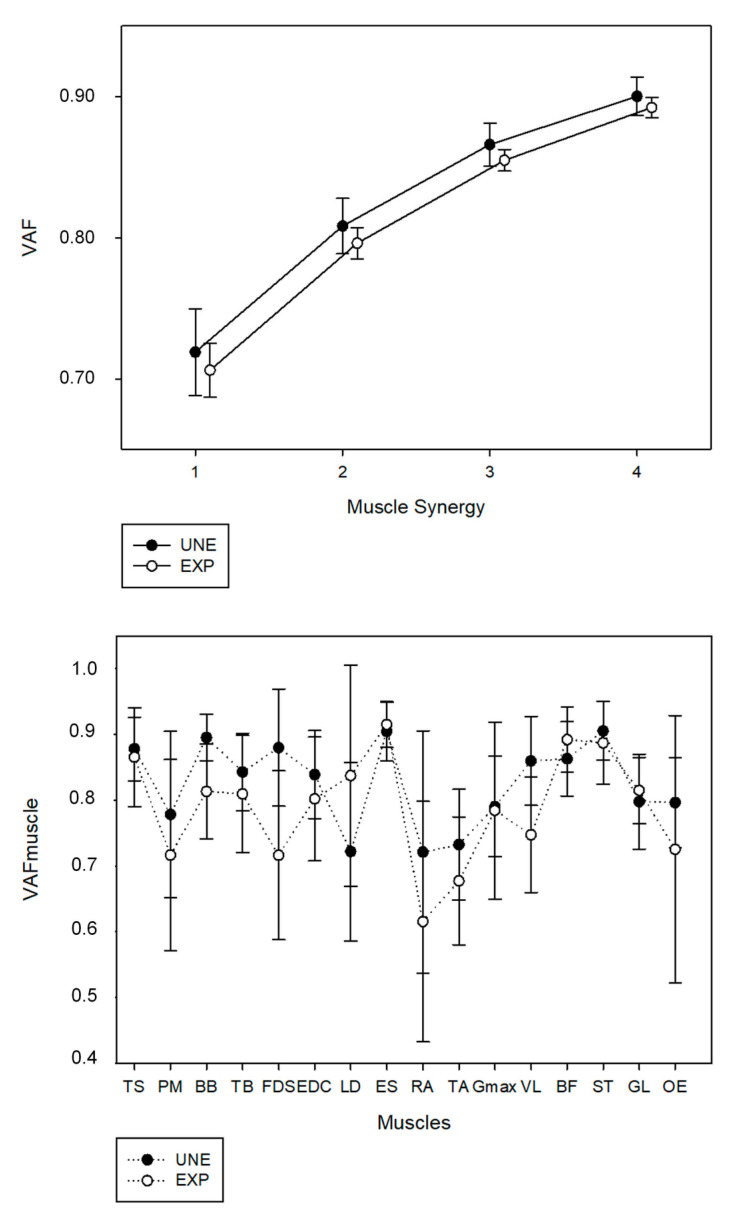
Top panel: Mean values of variance accounted for (VAF) relatively to the original extraction iteration of muscle synergies for unexperienced participants (UNE) and weightlifters (EXP); Down panel: mean values of variance accounted for each muscle (VAF_muscle_) regarding a three synergy-model for day one and two.

Using the described criteria to identify the number of muscle synergies, three muscle synergies were identified for all participants from both UNE and EXP groups. The three-extracted muscle synergies accounted for a similar VAF between EXP (85.5 ± 0.7%) and UNE participants (86.6 ± 1.5%), as represented in Figure 1. The fourth synergy did not account for the defined 5% of VAF, accounting only 3.7 ± 0.6% for EXP and 3.4 ± 0.3% for UNE. No statistical differences were found in VAF for the three extracted muscle synergies between groups (*p* = 0.133; d = 0.01).

For both groups, RA and TA accounted for less than 75% of VAF_muscle_, as well as PM, FDS and OE for EXP and LD for UNE. Significant differences in VAF_muscle_ of BB (*p* = 0.011; d = 0.30) and FDS (*p* = 0.014; d = 0.244) were found between groups. VAF_muscle_ of each muscle is presented in Figure 1.

For UNE group, muscle synergy #1 mainly represented the back and posterior muscles of the lower-limb (TS, LD, ES, Gmax, BF, ST, GL). The muscle synergy #2 mainly involved the upper-limb muscles (TS, BB, TB, FDS, EDC) and the muscle synergy #3 represented the final of the ascendant phase and involved mainly the trunk muscles (RA, OE, ES, PM), VL, and TA. For the EXP group, although the general composition of each synergy was similar, some differences were found. In synergy #1, the LD and ES presented a lower weighting than for UNE, while VL was encompassed mainly in this synergy. Synergy #2 showed to be composed by the same group of muscles in both groups. Finally, in synergy #3, the main muscles were ES and LD, while TA and VL did not present such relevance.

### 3.2. Intra-Group Variability

For each group, intra-group variability was assessed. Regarding the synergy’s synchrony, i.e., the lag time, no significant shifts were found in UNE or EXP groups. Additionally, all r_max_-values showed a strong correlation (range: 0.77–0.87). However, for muscle synergy vectors, just moderate values of correlation were found in both groups (range: 0.34–0.52).

For each group, individual EMG patterns were strongly correlated (0.73 < r_max_ < 0.92). However, for UNE, shifts were found in PM (*p* = 0.03; d = −0.32) and TA (*p* = 0.01; d = 0.39), and for EXP, shift were found also in PM (*p* = 0.01; d = −0.31), EDC (*p* = 0.01; d = 0.39), and VL (*p* = 0.02; d = −0.28).

### 3.3. Inter-Group Variability

**Figure 2:**
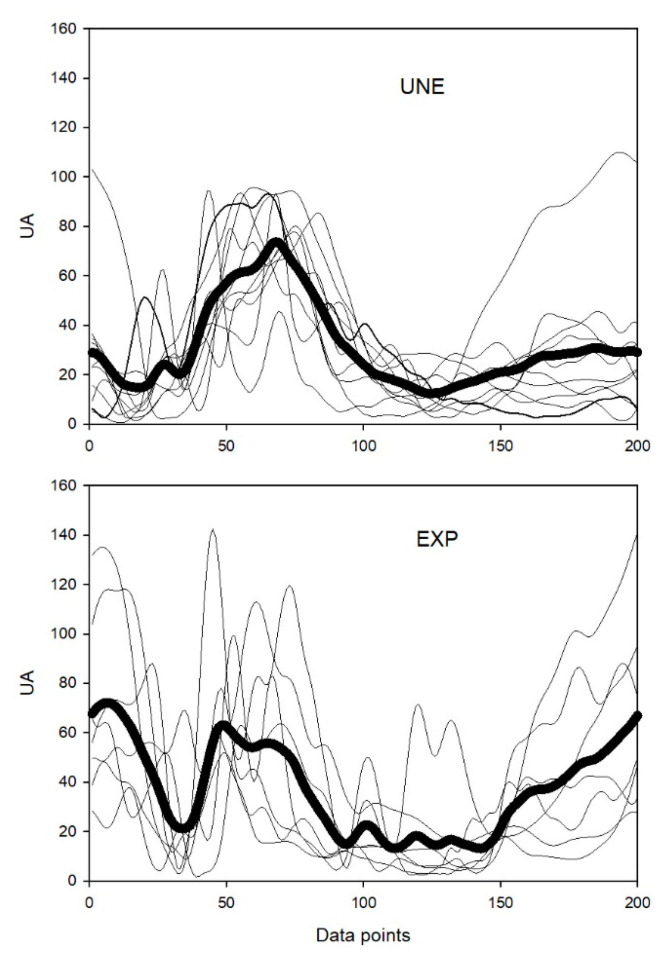
Inter-individual variability of Synergy #3 activation coefficients (UA—arbitrary units). Bottom panel corresponds to weightlifters’ group while top panel regards to unexperienced participants’ group. The thick black line represents the group mean, while the thin lines represent individual synergy activation coefficients.

**Figure 3:**
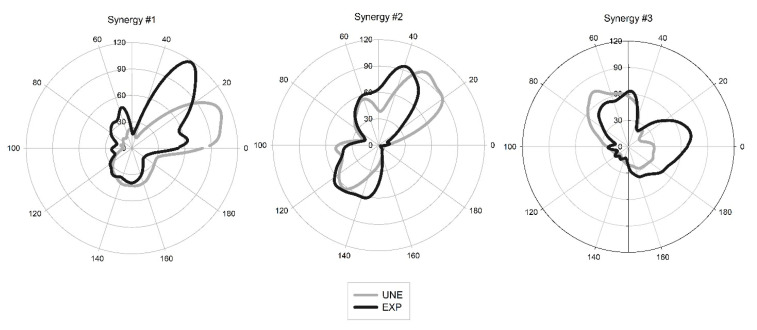
Averaged synergy activation coefficients of weightlifter (EXP) and unexperienced participant (UNE) groups. Left panel refers to Synergy #1, central panel to Synergy #2, and right panel to Synergy #3. The upper hemisphere of the graphs corresponds to the ascendant phase (0–100% of the power clean cycle), while the lower hemisphere corresponds to descendant phase (100–0% of the power clean cycle).

Comparing both groups intra-group variability, in synergy #1 and #2, no differences were found in r-values and r_max_-values corresponding to muscle synergy vectors and synergy activation coefficients, respectively. However, for synergy #3, a significant difference in r_max_-values was found (*p* = 0.001; d = 1.36), presenting the UNE group a higher correlation value (Figure 2). Regarding the inter-group analysis, significant shifts were found in the three extracted synergies, with synergy #1 (*p* < 0.001; d = −0.55) and #2 (*p* < 0.001; d = −0.66) being activated earlier for UNE and synergy #3 (*p* = 0.005; d = 0.34) being activated earlier for EXP (Figure 3).

**Figure 4:**
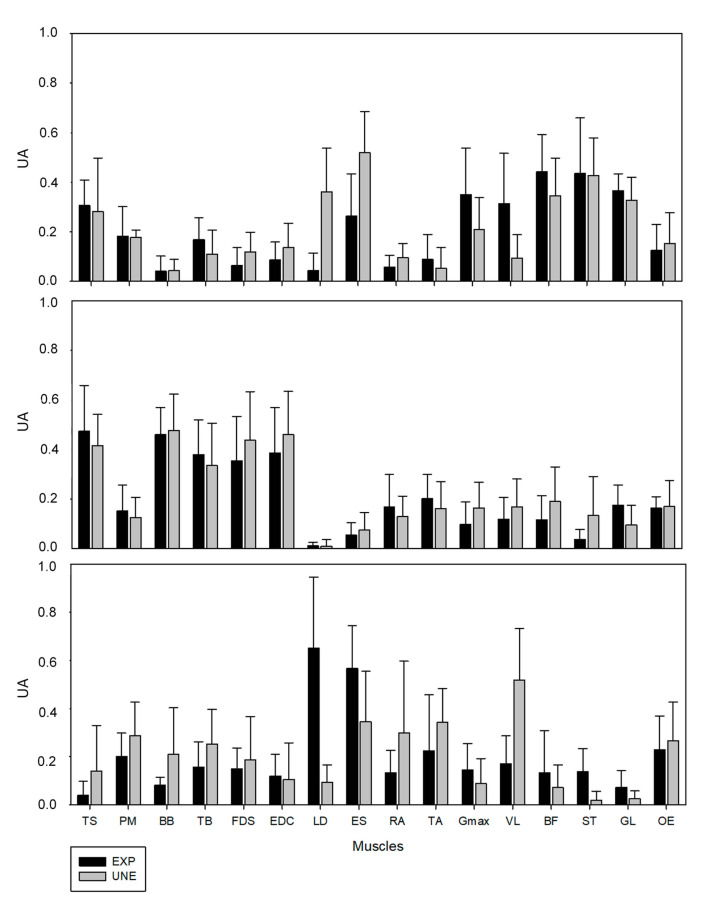
Averaged muscle synergy vectors of weightlifter (EXP) and unexperienced participant (UNE) groups (UA–arbitrary units). Top panel regards to Synergy #1, central panel to Synergy #2, and bottom panel to Synergy #3.

The similarity indexes of the synergy activation coefficients were high, presenting strong correlations (0.78, 0.88, and 0.76 for synergy #1, #2, and #3, respectively). However, muscle synergy vectors presented weak to moderate correlations (0.31, 0.53, and 0.10 for synergy #1, #2, and #3, respectively), as presented in Table 1 and Figure 4.

**Figure 5:**
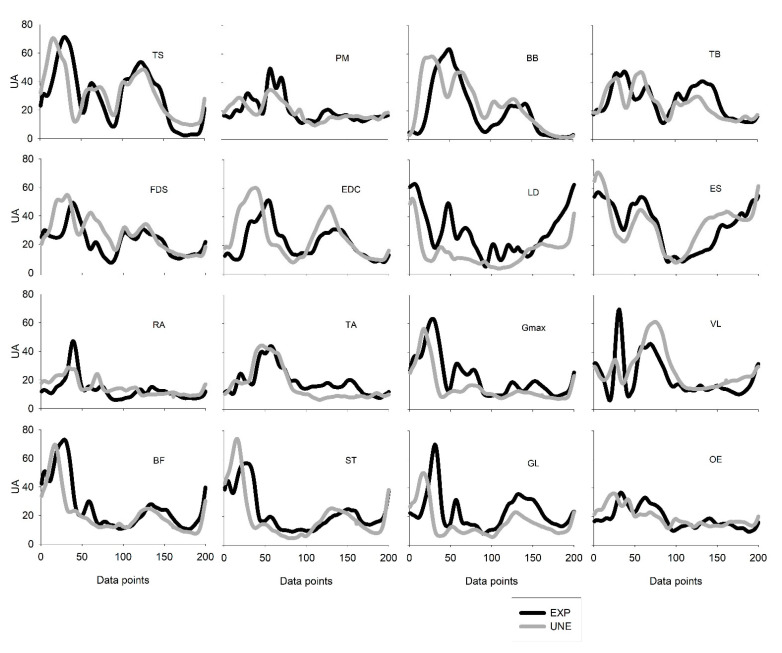
Averaged EMG envelopes (UA—arbitrary units) from 16 muscles obtained in weightlifters (EXP) and unexperienced participants during power clean cycle (200 time points).

Between groups, even though correlation-values were still strong (0.82 < r_max_ < 0.91), more temporal adjustments were found. UNE participants presented significant backward shifts in TS (*p* < 0.001; *d* = −0.95), BB (*p* < 0.001; *d* = −0.52), EDC (*p* < 0.001; *d* = −0.74), Gmax (*p* < 0.001; *d* = −0.82), BF (*p* < 0.001; *d* = −0.72), ST (*p* < 0.001; *d* = −0.74), GL (*p* < 0.001; *d* = −0.77), and OE (*p* < 0.001; *d* = −0.54). However, UNE participants presented a delayed activation of VL (*p* = 0.01; *d* = 0.30). Data concerning similarity values are presented in Table 2 and Figure 5.

### 3.4. Cross-Validation of Muscle Synergies

We assessed the similarity of muscle synergies between the two groups using the muscle synergy vectors extracted from untrained participants (average dataset of all the untrained participants) to reconstruct the EMG patterns of each untrained participant and each weightlifter. Muscle synergy vectors of the untrained participants explained 85.0 ± 2.0% of VAF for the untrained participants and 78.2 ± 4.9% of VAF for weightlifters (*p* = 0.009; d = 0.96). The difference between groups was higher than the variability within the untrained participants. From the 7 weightlifters, 4 presented VAF values <80% (range: 70.5–79,6%), which are defined by some authors as the threshold that determines the number of synergies [35]. All the untrained participants presented VAF values >80%.

## 4. Discussion

The aim of this study was to investigate and compare the neural strategies underlying the power clean exercise between experienced and unexperienced participants. While the shape of activation patterns of synergies presented strong correlations across participants of each group, the weightings of each muscle within the synergies were more variable. Comparing groups, the synergistic organization of muscle coordination was not profoundly affected by expertise. However, the three extracted synergies were shifted in time with the UNE group anticipating synergy #1 and #2 and delaying synergy #3. Nevertheless, the synergies presented similar shapes between groups, unlike muscle synergy vectors that were rather more variable, even considering the existing changes in composition of synergy #1 and #3.

The groups presented differences in weight regarding to the heavy weight classes of EXP participating in the study when compared with the UNE. Moreover, differences in the lifted load during the 5 RM test were expected since the EXP were specialized in the task and currently training. 

### 4.1. Muscle Synergies

For both groups, three muscle synergies were extracted based on the defined criteria of VAF. Previous studies have also shown muscle synergies to be robust across expert cyclists [19], rowers [20], gymnasts [21], and powerlifters [23] as well as untrained participants during rowing [20], pedaling [36], and bench pressing [23]. However, we should note that the cross-validation analysis revealed that, although the number of synergies was similar, the reconstructed EMG patterns of the EXP from the UNE dataset were significantly different. Thus, the difference between populations was higher than the variability within the UNE, which means that specific resistance training resulted in different neural strategies adopted by the EXP to perform the exercise.

A similar number of muscle synergies was observed in other complex motor tasks like rowing [20], breaststroke swimming [22] or backward giant swing [21]. However, the only two extracted muscle synergies in bench press [23] may be viewed as a result of the less complexity of the exercise [21]. 

It is interesting to note that differences in VAF_muscle_ were found in BB and FDS, two upper-limb muscles. This can possibly be explained by the unusual grip condition for the EXP. These individuals typically use lifting straps that allow them to focus only on the pulling action. To standardize the evaluation situation for both groups, all participants performed the power clean without lifting straps. This may be related with some observed variability at the grip level in EXP, that could possibly influence the VAF_muscle_.

### 4.2. Intra-Group Variability

For each group, we found generally similar values of correlation for the synergy temporal component. For UNE and EXP, the pairwise analysis did not reveal significantly different timing characteristics of the synergies. This means that the temporal activation of each synergy within each group was consistent across subjects. Moreover, for each group, the shape of the synergies was generally similar, exhibiting little intra-group variability. However, significant differences between correlation values of synergy #3 were found between groups, revealing that UNE were less variable. This is in line with a previous study in bench press showing that powerlifters utilized specific motor strategies well adapted to individual anthropometry and muscle architecture, thereby promoting a better performance in the task [23]. Additionally, another study showed that although activation coefficients of two of the three extracted synergies in elite rowers were less variable than in untrained subjects, the third synergy of rowers presented great variability [20]. This may suggest that experts modulate and adapt this synergy to their specific characteristics through training. It is also possible that the training method may influence this modulation.

Regarding the spatial component of synergies, the muscle synergy vectors, more variability was found within each group. This supports the existing data from other tasks showing that muscle vectors are more variable than activation coefficients for EXP in backward giant swing [21] and pole vaulting [25], and for EXP and UNE in bench press [23] and in rowing [20]. In both groups, the correlation between muscle vectors was moderate for the three extracted synergies. This may represent the musculoskeletal redundancy of motor behavior, which allows the central nervous system to adopt infinite solutions to perform a task [37], meaning that although the activation of the synergies is consistent in time, the muscle weightings may vary.

### 4.3. Inter-Group Variability

We observed that during power clean, the synergy #3 of EXP participants had two peaks, contrary to UNE’s synergy #3 that had only one peak (Figure 2). This difference may be related with the integration of synergy #1 and #3 of EXP in the final of descendant phase and in the beginning of ascendant phase. While in UNE, the synergy #3 probably relates only the knee extension to reach the stand position, in EXP the third synergy pattern shows a second increasing moment whose peak coincide with the beginning of activation of the first synergy. With the existence of this peak, synergies #1 and #3 for EXP present some level of coactivation, which is not observed for UNE. With this coactivation, the synergy #1 of UNE seems to be divided into the synergy #1 and the first peak of synergy #3 of EXP. This fractionation results into an incorporation of the VL for the EXP in synergy #1, while the ES and the LD compose mainly synergy #3. The fractionation of muscle synergies has been observed in stroke chronically-affected patients [38]. In this study, patients presented fractionations of some muscle synergies when comparing the affected with the unaffected-arm, and the fractionation was more evident in patients with longer post-stroke duration, which may have been an adaptive process triggered in response to the poststroke impairment. In our case, the fractionation observed in EXP when compared with UNE, may be related with an adaptation in intermuscular coordination developed through training. This adaptation may have provided a more flexible control, mainly, of the lower-limb and back muscles while preparing and starting the implementation of the movement. 

As expected, muscle vectors presented low values of correlation. With the exception of synergy #2 that showed to be less variable in composition, synergy #1 and #3 presented weaker correlations. Regarding synergy #2, this possibly means that UNE are relying on intrinsic synergies already used in similar motor tasks, since the variation within both groups was approximately the same as between-groups. For synergy #1 and #3, the weaker correlations may be associated to modulation of muscle synergies by the EXP. This is in line with previous studies finding that specific strategies in response to unexpected external perturbations were developed by elite hockey players [17], and that a new synergy was incorporated in elite badminton players during the smash shot [24]. Moreover, after four weeks of sensorimotor training, untrained individuals modified modular organization during landing [27], while five weeks of bench press training provided a more individualized motor strategy to the individuals [26].

Regarding temporal parameters, the three extracted synergies revealed time shifts between both groups. Despite a strong correlation in activation coefficients observed between the two groups, synergy #1 and #2 exhibited a significant backward time shift in UNE. This result is accordance with previous study referring to breaststroke swimming, showing that one of the extracted synergies was anticipated in novices when compared to experts [22]. The backward shift observed for synergy #1 is mainly related with the lower-limb muscles. The UNE showed to present some variability between sets in temporal parameters of the BF and GL. These muscles are represented in this synergy, and the time adjustments may be related with variations in the starting position of the ascendant phase [31]. Additionally, significant shifts were found for individual EMG patterns, which means that all the muscles that contribute to lower-limb triple extension (with exception of the VL) present a backward shift for UNE. Thus, UNE may anticipate the activation of the synergy to compensate the initial angular position of the hip, eventually more closed than for EXP (with deeper squat position and with great trunk forward inclination). For synergy #2, it is observed the greater shift. UNE present an activation of synergy involving the upper-limb muscles anticipated in relation to EXP. These muscles present, generally, an earlier activation along the cycle for UNE, and it may be related with the early flexion of the upper-limbs when compared to EXP. Namely for the UNE, the scapular elevation/upper rotation and the forearm flexion, by TS and BB, respectively, while the lower-limbs are not completely extended may justify the anticipation of synergy #2. Nevertheless, it is interesting to note that this synergy presented the higher correlation value. This means that despite a considerable shift in time, the shape of the activation pattern does not vary between groups. Thus, previous coordination strategies utilized in other tasks may be shared between different behaviors [39], and this contribute may increase motor performance during a subsequent task, in this case the power clean. Relatively to synergy #3, the UNE delayed its activation, despite that the composition of this synergy being slightly different between EXP and UNE, UNE presented a forward shift regarding the final of the ascendant phase. This may be explained by the pattern of VL, that during triple extension of lower limbs presented a delayed activation.

Although the present study has a small sample size, most of the significant differences found regarding the muscle synergy components and individual EMG patterns presented a moderate to large effect size (Cohen’s *d* above 0.5), going beyond the variability of the data regarding muscle synergy extraction during the power clean [31]. The between-group differences observed in this study suggest the need for further research regarding strength training adaptations, including OWL exercises. This would possibly allow to assess how unexperienced participants could modify coordination over time. Thus, integrate untrained individuals to a training protocol should be the next step to demonstrate how these adaptations of neural strategies to perform complex movements tend to evolve, and what are the phases of the training process that should be considered when the objective is to promote intermuscular adaptations. Moreover, as a next step, adding kinematic data could possibly provide information about the muscle activation during the movement, corroborating the results obtained through the EMG analysis.

## 5. Conclusions

The present study showed that some adjustments in movement coordination may emerge with training, namely in what concerns which muscles are activated during the movement and their precise timings of activation. Additionally, the findings of this study indicate that the unexperienced individuals present similar strategies of muscle coordination performing a strength training complex task that could be associated to similar performance errors. Thus, it should be recommended that during the learning phase of the movement, namely in sports where weightlifting movements are just a method to improve strength and power (like in team sports) and technique is not always a priority, athletes should attempt to delay the hip extension, as well as the upper limbs flexion, in order to approximate the muscle patterns to those observed in weightlifters. It is also important that participants are able to activate and coordinate the lower-limb and back muscles during the initial phase of the exercise, providing a more flexible control of the movement. 

## Figures and Tables

**Table 1:** Intra-group and inter-group similarity values (r) of muscle synergy vectors of weightlifters (EXP) and unexperienced participants (UNE).

	Intra-Group		Inter-Group
	UNE	EXP	
#1	0.52 ± 0.22	0.40 ± 0.27	0.31 ± 0.30
#2	0.51 ± 0.23	0.52 ± 0.13	0.53 ± 0.18
#3	0.34 ± 0.23	0.47 ± 0.34	0.10 ± 0.26

**Table 2:** Intra-group and inter-group similarity values (r_max_) and lag times (%) of synergy activation coefficients and individual EMG profiles of weightlifters (EXP) unexperienced participants (UNE). Bold values represent statistical differences from zero and Cohen’s d above 0.8.

	Intra-Group|UNE				Intra-Group|EXP				Inter-Group			
	% lag	*p*	*d*	r_max_	% lag	*p*	*d*	r_max_	% lag	*p*	*d*	r_max_
**Individual EMG Profiles**												
**TS**	−0.03 ± 1.60	0.53	−0.09	0.88 ± 0.05	1.26 ± 3.15	0.17	0.17	0.89 ± 0.05	−2.60 ± 2.73	**<0.001**	**−0.95**	0.86 ± 0.06
**PM**	−2.63 ± 7.73	**0.03**	−0.32	0.86 ± 0.06	2.29 ± 3.26	**0.01**	0.31	0.87 ± 0.04	0.79 ± 7.13	0.88	0.02	0.85 ± 0.06
**BB**	0.29 ± 5.56	0.29	0.16	0.89 ± 0.07	1.62 ± 3.63	0.06	0.25	0.92 ± 0.04	−3.94 ± 6.49	**<0.001**	−0.52	0.86 ± 0.04
**TB**	−0.49 ± 8.87	0.68	−0.06	0.89 ± 0.04	0.69 ± 2.20	0.26	0.14	0.91 ± 0.04	0.20 ± 6.94	0.36	−0.11	0.88 ± 0.05
**FDS**	0.86 ± 8.26	0.24	0.18	0.86 ± 0.05	−0.95± 2.08	0.06	−0.23	0.82 ± 0.07	0.08 ± 9.35	0.72	−0.04	0.84 ± 0.06
**EDC**	−0.19 ± 2.96	0.70	−0.06	0.88 ± 0.04	3.74 ± 5.24	**0.01**	0.39	0.85 ± 0.06	−6.47 ± 6.57	**<0.001**	−0.74	0.86 ± 0.06
**LD**	0.00 ± 0.00	1.00	0.00	0.82 ± 0.08	−4.03 ± 15.1	0.47	−0.09	0.73 ± 0.12	0.27 ± 11.43	0.38	−0.10	0.76 ± 0.09
**ES**	0.00 ± 0.00	1.00	0.00	0.91 ± 0.03	0.13 ± 0.50	0.32	0.12	0.91 ± 0.03	−0.16 ± 1.51	0.66	−0.05	0.89 ± 0.05
**RA**	−4.07 ± 10.2	0.26	−0.33	0.84 ± 0.07	−2.57 ± 4.49	0.05	−0.26	0.83 ± 0.09	3.39 ± 11.51	0.28	0.13	0.84 ± 0.08
**TA**	1.41 ± 3.57	**0.01**	0.39	0.89 ± 0.05	1.21 ± 4.00	0.19	0.17	0.89 ± 0.05	−0.77 ± 3.79	0.09	−0.20	0.87 ± 0.07
**Gmax**	0.29 ± 3.22	0.55	0.09	0.88 ± 0.06	−5.10 ± 14.5	0.14	−0.18	0.83 ± 0.11	−8.83 ± 13.8	**<0.001**	**−0.82**	0.83 ± 0.08
**VL**	−0.40 ± 5.06	0.25	0.17	0.91 ± 0.05	−0.36 ± 0.64	**0.02**	−0.28	0.89 ± 0.04	2.29 ± 6.16	**0.01**	0.30	0.84 ± 0.07
**BF**	0.11 ± 0.87	0.31	0.15	0.89 ± 0.04	−0.21 ± 1.15	0.48	−0.09	0.89 ± 0.04	−3.01 ± 2.86	**<0.001**	−0.72	0.87 ± 0.05
**ST**	−0.09 ± 0.83	0.33	−0.15	0.90 ± 0.05	0.24 ± 1.82	1.00	0.00	0.86 ± 0.06	−3.31 ± 2.73	**<0.001**	−0.74	0.83 ± 0.05
**GL**	−0.49 ± 11.2	0.14	−0.22	0.83 ± 0.08	−0.12 ± 2.56	0.08	−0.22	0.88 ± 0.05	−5.74 ± 3.57	**<0.001**	−0.77	0.82 ± 0.06
**OE**	0.28 ± 5.13	0.72	0.05	0.85 ± 0.05	−4.67 ± 12.8	0.31	−0.13	0.86 ± 0.06	−6.94 ± 10.9	**<0.001**	−0.54	0.85 ± 0.06
**Synergy Activation Coefficients**												
**#1**	0.06 ± 0.91	0.72	0.05	0.86 ± 0.07	3.21 ± 8.09	0.11	0.35	0.86 ± 0.06	−2.46 ± 18.7	**<0.001**	−0.55	0.87 ± 0.08
**#2**	0.79 ± 7.87	0.29	0.16	0.87 ± 0.06	0.57 ± 3.56	0.48	0.16	0.89 ±0.06	−4.60 ± 5.71	**<0.001**	−0.66	0.90 ± 0.06
**#3**	−2.51 ± 17.2	0.68	−0.06	0.86 ± 0.08	0.45 ± 14.25	0.55	−0.13	0.77 ± 0.10	1.86 ± 17.39	**0.01**	0.34	0.87 ± 0.08

## Data Availability

No new data were created or analyzed in this study. Data sharing is not applicable to this article.
